# Impact of Diet on Inflammatory Bowel Disease Symptoms: An Adolescent Viewpoint

**DOI:** 10.1093/crocol/otaa084

**Published:** 2020-11-12

**Authors:** Megan T Zangara, Natalie Bhesania, Wei Liu, Gail A M Cresci, Jacob A Kurowski, Christine McDonald

**Affiliations:** 1 Department of Molecular Medicine, Cleveland Clinic Lerner College of Medicine of Case Western Reserve University, Cleveland, Ohio, USA; 2 Department of Inflammation and Immunity, Lerner Research Institute, Cleveland Clinic, Cleveland, Ohio, USA; 3 Division of Pediatric Gastroenterology and Nutrition, University of Mississippi Medical Center, Jackson, Mississippi, USA; 4 Department of Quantitative Health Sciences, Lerner Research Institute, Cleveland Clinic, Cleveland, Ohio, USA; 5 Department of Medicine, Cleveland Clinic Lerner College of Medicine of Case Western Reserve University, Cleveland, Ohio, USA; 6 Department of Pediatric Gastroenterology, Hepatology & Nutrition, Cleveland Clinic, Cleveland, Ohio, USA

**Keywords:** adolescents, diet, inflammatory bowel disease, survey

## Abstract

**Background:**

Dietary modification shows promise as therapy in inflammatory bowel disease (IBD); however, it is unknown whether adolescents are interested in a dietary approach.

**Methods:**

Cross-sectional survey of adolescents with IBD ages 14–21 on disease knowledge, dietary habits, and perceptions of diet therapy.

**Results:**

A total of 132 subjects (48.5% female), mean age of 17.8 years and median disease length of 5 years (range 0, 16), completed the survey. Diet was perceived as a symptom trigger by 59.8% of subjects, and 45.4% had tried using diet as a treatment for symptom resolution, often without physician supervision and with limited success. Subjects experiencing active disease symptoms as determined by Manitoba IBD Index were more likely to be currently modifying their diet compared to subjects without active disease symptoms (odds ratio = 4.11, confidence interval = 1.58, 10.73, *P* = 0.003).

**Conclusions:**

Adolescents with IBD perceive a relationship between diet and disease symptoms and are interested in dietary modification as a symptom management option.

## INTRODUCTION

Inflammatory bowel disease (IBD) is a chronic inflammatory condition caused by a combination of multiple risk factors, resulting in relapsing inflammation of the gut.^1^ IBD can present at any age; however, the peak age of onset is between 15 and 35 years of age, with approximately 1 quarter of all IBD patients diagnosed before the age of 20.^2,3^ Traditional therapeutic strategies involve immunosuppressive drugs to control the inflammation caused by aberrant immune activation. However, IBD is multifactorial in etiology, and these types of treatments do not directly address 2 major risk factors for disease development: alterations to the intestinal microbiome and consumption of a Western diet.^4–7^ Diet and the microbiome play into and off of one another in both the development and progression of disease, making them ideal targets for new lines of induction and maintenance therapies.^5–7^ Evidence from etiological and interventional studies suggest that diet and microbiome-focused therapeutics may be most beneficial in adolescent and young adult IBD patients.^8–12^

Adolescence is a challenging time for IBD patients due to increased social pressures, desire for independence, and the shifting of disease management responsibility from the parent to the child.^13^ There is a high rate of treatment noncompliance (75%–85%) in adolescents, and noncompliance is a major risk factor for disease relapse, acute hospitalization, and surgery.^13^ During this time of their life, it is critical that IBD patients understand the nature of their disease and the importance of treatment adherence to effectively manage their disease and improve their quality of life.

As food is a large aspect of social experiences, compliance with a strict dietary regimen to manage disease symptoms may be difficult for adolescents. This difficulty is compounded by the fact that IBD can cause psychological stress centered on the topics of food and nutrition.^14^ Therefore, a greater understanding of the factors that adolescents view as barriers to following treatment plans and an evaluation of the effectiveness of current platforms used to communicate disease information are needed to design successful strategies for disease management in adolescent IBD patients. The aim of the current study was to examine the knowledge, perceptions, and attitudes of adolescents and young adults with IBD on the relationships between disease, diet, and dietary modification for symptom management.

## MATERIALS AND METHODS

### Subject Recruitment

We conducted a cross-sectional survey of adolescent IBD patients ages 14–21 who presented for routine care in the Department of Pediatric Gastroenterology at Cleveland Clinic Children’s Hospital between April and September 2018. The study protocol was approved by the Cleveland Clinic Pediatric Institute Research Committee and Institutional Review Board. Inclusion criteria for enrollment in the study were a diagnosis of IBD at least 3 months prior to enrollment and parent/legal guardian permission if under the age of 18 years. Patients were excluded if they were unwilling to participate or unable to complete the survey without assistance.

### Survey Design

**Figure 1.**
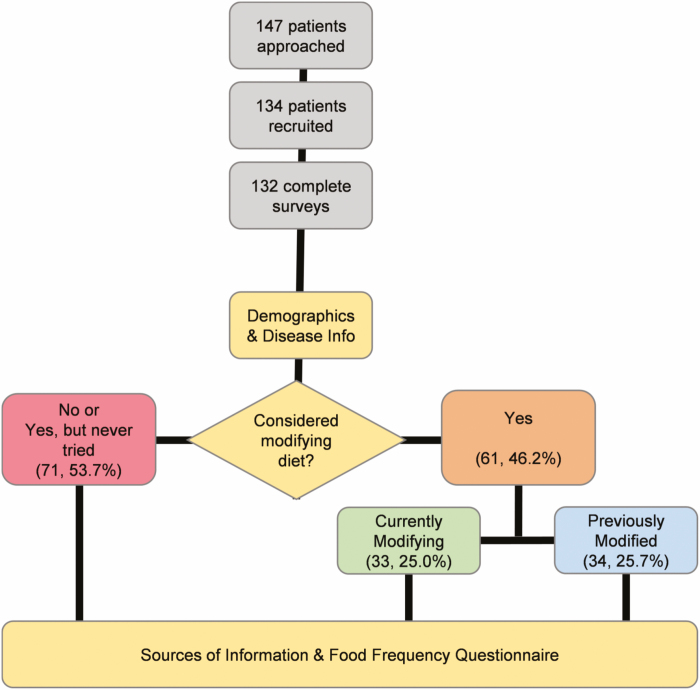
Branching pattern of survey design, displaying how subjects were divided into 3 subgroups used for analysis of diet perceptions. All subjects answered questions about demographics, disease information, and sources of information, as well as completed a food frequency survey (yellow boxes). The 3 subgroups were determined by self-reported diet utilization: never tried (red box), currently trying (green box), and previously tried (blue box).

The survey was designed based on published and validated patient surveys pertaining to diet,^15^ studies of adolescent IBD patients,^16,17^ the SF-36 Health Survey,^18^ the Manitoba IBD Index (MIBDI) survey,^19^ and in consultation with 2 pediatric gastroenterologists (J.A.K. and N.B.) and a dietitian with expertise in IBD (G.A.M.C.). The survey was converted to an electronic format for administration and the data were collected using Research Electronic Data Capture (REDCap) survey tools on an electronic tablet. The questions were formatted utilizing a combination of check boxes, Likert-type rating scales, and short answer responses. The survey was made with a branching design, with the survey diverging on questions related to usage of dietary modifications to manage IBD symptoms (Fig. 1). Participants answered between 60 and 100 questions and average completion time was 15 minutes.

The survey incorporated questions to determine adolescent perceptions of diet in relationship to their disease, the prevalence of subjects currently attempting a diet, willingness to initiate and adhere to dietary modification, and factors that influence willingness to modify their diet. Dietary modifications listed in the survey were selected based on literature searches of recently published clinical trials and in consultation with J.A.K. and G.A.M.C. Subjects were given 2 additional diet options: “food exclusion” and “other.” Food exclusion diets were defined as a dietary modification that does not conform to the parameters of the established diets listed in the survey, but was a purposeful removal of 1 or several foods that are believed by the subject to aggravate their IBD. “Other” diets were anything they did not think matched with the other provided categories. Information about the subject’s current diet was collected as part of the survey using a food frequency questionnaire (Supplementary Data Content 1). Using fillable text boxes, subjects were asked to identify food(s), if any, that they perceived altered their IBD symptoms.

Additionally, the survey collected the subject’s demographic information, and information regarding their knowledge of disease diagnosis, disease activity, history, and medications. Disease activity was categorized using the MIBDI,^19^ which assesses disease activity based on a patient’s 6-month recall of symptom persistence. For all completed surveys, clinical data from the electronic medical record (EMR) were collected regarding disease diagnosis, disease history, and current treatments. Subject responses were compared with the same disease information recorded in the EMR for accuracy. The subjects were also asked where they obtained their IBD management information and what resources they found most useful or would benefit from being available to help them understand their disease and to identify avenues to improve their knowledge. The subject’s responses are abbreviated as SR and electronic medical record reported information on disease and diet is abbreviated as EMR-R.

### Data Analysis

Subject demographics, severity of disease, dietary influences, EMR and comparable results in surveys, and perceptions on diet usage were described by medians and ranges for continuous variables, and counts and percentages for categorical variables. Questions pertaining to diet usage were analyzed in subcohorts (Fig. 1). The first subcohort of subjects self-reported currently utilizing purposefully modified diets to manage IBD symptoms. The second subcohort of subjects self-reported having tried purposefully modified diets to manage IBD symptoms in the past. The third subcohort of subjects never considered changing their diet as part of treatment or who only considered changing their diet, but never tried.

All subjects were given a list of disease descriptors used to describe IBD characteristics. They were asked to select all descriptors they believed had been used by their physician to describe their disease, to the best of their knowledge. To assess this knowledge, a point was awarded for every term correctly marked (indicating the term had been used) or left blank (indicating it was not to used). Receiving 75%–100% of total possible points was considered good, 40%–74% fair, and less than 40% poor knowledge. Lists of medications and supplements (eg, vitamins/minerals, herbals, oral-liquid drinks) commonly prescribed to IBD patients were similarly presented on the survey and analyzed in the same manner.

Data analysis was performed using SAS version 9.4 (SAS Institute, Cary, NC) and R version 3.50 (Vienna, Austria) software. McNemar’s test and the exact symmetry test were used to compare results between physician-filled EMRs (EMR-R) and subject-reported surveys (SR), as well as the source of information related to IBD and diet among current diet users. Gwet agreement coefficients (AC1)^20^ and 95% confidence intervals (CIs) were used to measure agreement between SRs and physician-filled EMR for outcomes of interest. Pearson chi-square test was used to compare MIBDI disease activity between diet cohorts and compare diet modification between levels of medication knowledge. All tests were 2-tailed with an alpha of 0.05.

## RESULTS

### Study Population

One hundred forty-seven adolescents and young adults with IBD were approached for the study of which 132 subjects completed the survey (Fig. 1), resulting in a response rate of 89.8%. Of the 132 subjects, 82.6% had Crohn’s disease, 13.6% ulcerative colitis, and 3.8% indeterminate colitis, with a median disease length of 5 years (Table 1). Mean age at enrollment was 17.8 years (14, 21), and median age at diagnosis was 13 years.

**Table 1.** Demographics of Study Population

Characteristic	SR	EMR-R	Gwet AC1
Male (n, %)	68 (51.5)	—	—
Female (n, %)	64 (48.5)		
Race (%)			
American Indian/Alaskan Native	0.76	—	—
Asian	1.5		
Black/African American	9.1		
Hispanic/Latino	6.1		
White	86.4		
Prefer not to say	2.3		
Age at enrollment, years (mean, [range])	17.8 [14, 21]	—	—
Age at diagnosis, years (median, [range])	13 [4, 19]	13 [2, 18]	—
Length of disease, years (median, [range])	5 [0, 18]	5 [0, 16]	—
MIBDI score (n, %)			
Inactive	61 (45.8)	—	—
Active	71 (54.2)		
IBD subtype (n, %)			
Crohn’s disease	112 (83.6)	109 (82.6)	0.94 (0.90, 0.98)
Ulcerative colitis	20 (14.9)	18 (13.6)	
Unclassified/indeterminate colitis	2 (0.14)	5 (3.8)	
Disease location (n, %)			
Small intestine only	37 (27.8)	20 (15.2)	0.33 (0.22, 0.46)
Large intestine only	26 (19.5)	28 (21.2)	
Both small and large intestine	44 (33.1)	84 (63.6)	
Don’t know	26 (19.5)	—	
Medications (n, %)			
Antibiotics	6 (4.5)	5 (3.8)	0.91 (0.85, 0.96)
Aminosalicylates	25 (18.9)	49 (37.1)	0.62 (0.48, 0.75)
Corticosteroids	12 (9.1)	21 (15.9)	0.82 (0.73, 0.90)
Immunomodulators	51 (38.6)	67 (50.8)	0.58 (0.44, 0.72)
Biologic therapies	78 (59.1)	88 (66.7)	0.80 (0.70, 0.90)
None	4 (3.0)	2 (1.5)	0.97 (0.94, 1.00)
Other	16 (12.1)	0 (0)	0.86 (0.79, 0.93)
Don’t know/don’t remember	9 (6.8)	—	—
Supplements (n, %)			
Iron	15 (11.4)	19 (14.4)	0.73 (0.62,0.83)
Folic acid/folate	15 (11.4)	41 (31.1)	0.61 (0.48, 0.75)
Vitamin D	33 (25.0)	73 (55.3)	0.037 (−0.14, 0.22)
Vitamin B12	6 (4.5)	0 (0)	0.95 (0.91, 0.99)
Fiber	3 (2.3)	0 (0)	0.98 (0.95, 1.00)
Multivitamin	23 (17.4)	19 (14.4)	0.77 (0.67, 0.87)
Probiotics	16 (12.1)	11 (8.3)	0.84 (0.76, 0.92)
None	85 (64.4)	24 (18.2)	−0.044 (−0.22, 0.14)
Other	10 (7.6)	10 (7.6)	0.89 (0.83, 0.96)
Don’t know/don’t remember	1 (0.76)	—	—

SR and EMR-R data are both reported when applicable. Gwet AC1 (95% CI) were used to measure agreement between SRs and medical record data.

**Figure 2.**
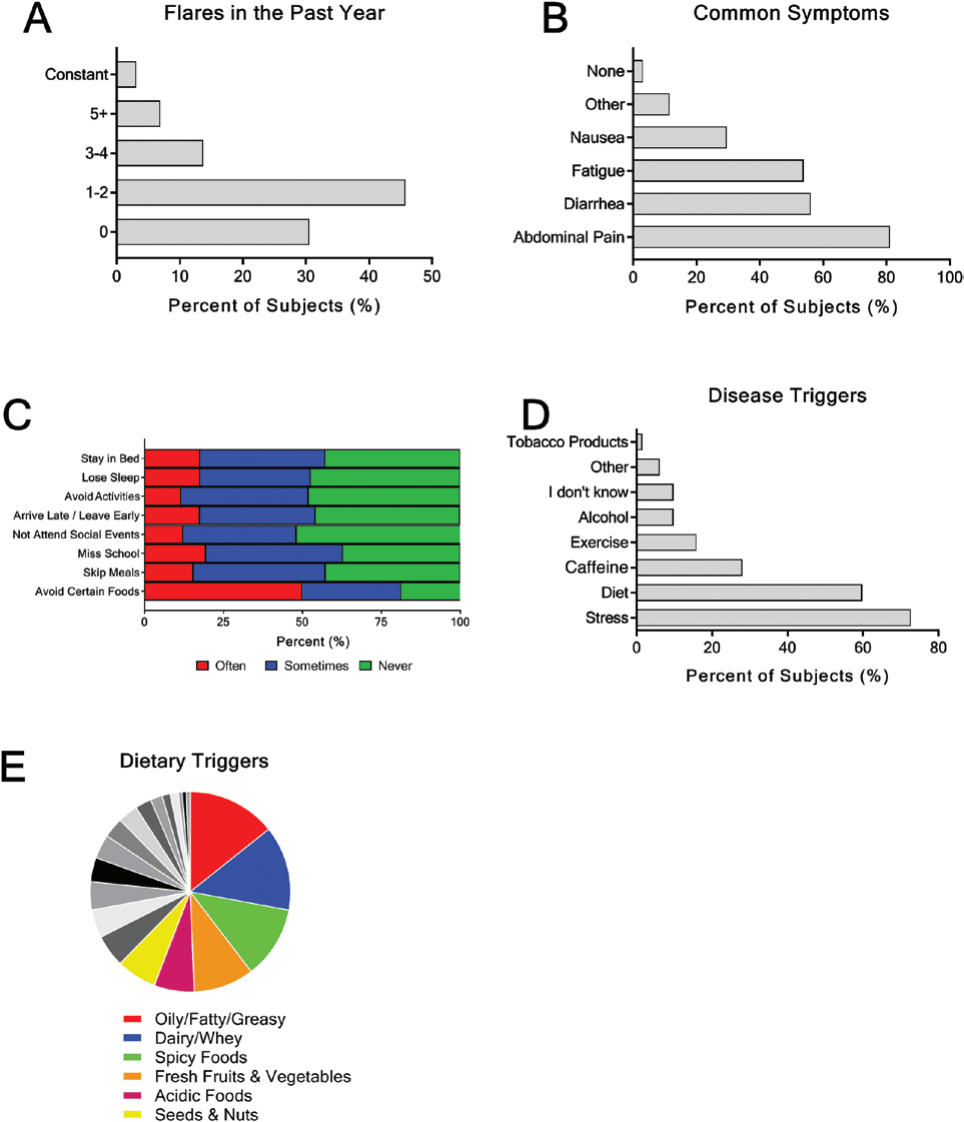
Disease activity and perceived triggers. A, Estimated number of disease flares experienced in the past year. B, Symptoms commonly experienced. C, How often IBD-related factors interfere with different aspects of daily life. D, Perceived potential disease symptom triggers. E, Subjects who selected diet as a potential trigger were provided a fillable text box to list food items they thought trigger their disease. Individual items were grouped by likeness; the top 6 categories are provided. Remainder of categories (grayscale sections) can be found in Supplementary Data Content 2.

The MIBDI was active (constant to occasional symptoms) in just over half (54.2%, n = 71) of subjects (Table 1). Most recalled experiencing at least 1 disease flare-up in the last year with abdominal pain, fatigue, and diarrhea identified as the most common symptoms experienced (Figs. 2A, B). Subjects reported that these symptoms disrupted their daily life, with 51.5% (n = 67) often avoiding certain foods and 15.3% (n = 20) skipping meals (Fig. 2C).

### Dietary Habits

Diet was identified as a symptom trigger by 59.8% (n = 79) of subjects, second only to stress (72.7%, n = 96) (Fig. 2D). A wide range of food items were reported as potential culprits. Similar to previous reports,^21–26^ oily and fatty foods, dairy, spicy foods, fresh fruits and vegetables, seeds and nuts, and acidic foods were the most frequently identified food categories, encompassing 65.2% of identified dietary triggers (Fig. 2E and Supplementary Data Content 2).

When asked about diet modifications to manage their IBD symptoms, just under half (45.4%, n = 60) of subjects had previously (25.8%, n = 34) or were currently (25.0%, n = 33) modifying their diet, with 7 subjects falling into both the previous and current diet modification categories. Subjects experiencing active disease symptoms as determined by MIBDI were more likely to be currently modifying their diet compared to subjects without active disease symptoms (odds ratio = 4.11, CI = 1.58, 10.73, *P* = 0.003). When asked which specific diet(s) they were currently utilizing or had tried in the past, subjects identified a wide variety of dietary modifications (Table 2). A food exclusion diet and a gluten-free diet were the most frequently previously utilized diets (n = 11 each), and a food exclusion diet was also the most currently utilized diet (n = 12). For all 3 top dietary interventions reported by subjects to be utilized, there was no dietary change recorded by the managing physician or dietitian (all *P* < 0.01) (Table 2).

**Table 2.** Diets Utilized by Subjects

	Current Dietary Modification			Prior Dietary Modification		
Diet	SR	EMR-R	*P*	SR	EMR-R	*P*
Food exclusion	12	0	**<0.001**	11	0	**0.001**
Gluten free	7	1	0.07	11	0	**0.001**
Low fiber	7	16	**0.04**	4	17	**0.01**
IBD Anti-Inflammatory	4	0	0.13	4	0	0.13
Enteral nutrition	3	0	0.25	8	2	**0.03**
Other	3	0	0.25	3	2	>0.99
Low fat	2	0	0.5	5	0	0.06
Low-FODMAP	2	2	>0.99	2	1	>0.99
Mediterranean	2	0	0.5	0	0	—
Specific carbohydrate	2	1	>0.99	2	1	>0.99
High fiber	1	1	0.99	2	0	0.5
Paleo	0	0	—	3	0	0.25

Subjects were presented a list of potential diet therapies, and ask to select all that they were currently utilizing, or had previously attempted. EMR notes were surveyed for mention of discussions about specific diets. The exact test of symmetry was used to determine significance of differences in reporting (α = 0.05). Comparisons that reached statistical significance are in bold text.

SR, subject reported.

**Figure 3.**
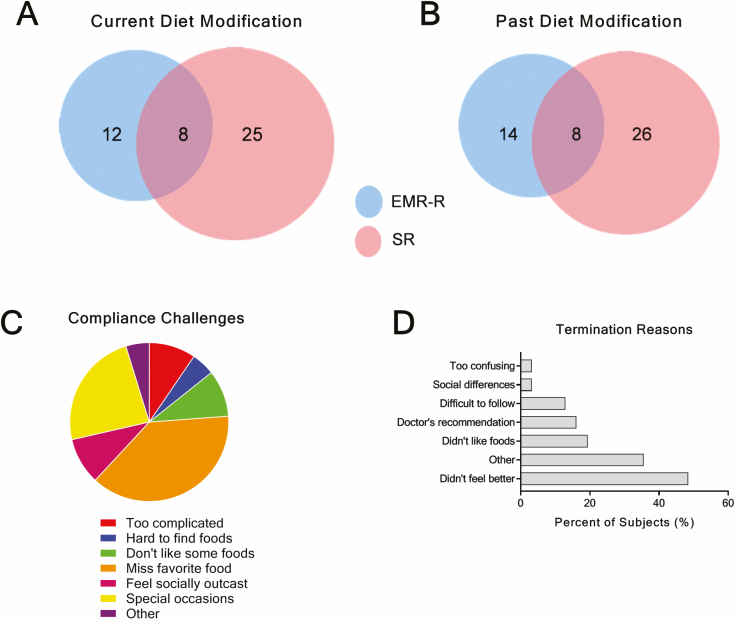
Dietary modification recording discrepancies, compliance challenges, and reasons for termination. A and B, SR diet usage (pink) EMR-R diet recommendations (blue), and number of subjects with both SR and EMR-R diet usage reported (purple). C, The top perceived reasons for compliance difficulty.

Overall, subjects reported following a diet significantly more often than documented in EMR-R (25.0% vs. 15.0%, *P* = 0.033), with 72% agreement between SR and EMR-R on current status of diet modification (AC1 = 0.59, CI = 0.45, 0.73). Of the 33 subjects who reported currently modifying their diet, only 8 (24.2%) were recommended to modify their diet by their physician, nurse, or dietitian (Fig. 3A). The converse was also observed with EMR-R recommended diet therapy recorded for 12 subjects (MIBDI 33% active, 66% inactive) without concomitant SR compliance (Fig. 3A). Similar findings were observed in responses from 34 subjects that reported previous dietary modification; only 8 (23.5%) had documented recommendations from their physician (AC1 = 0.43, CI = 0.25, 0.61) (Fig. 3B). Likewise, in this prior dietary modification group, an additional 14 subjects were previously recommended to modify their diet, but did not comply (Fig. 3B).

Twenty-one (70.0%) of subjects currently modifying their diet found dietary protocol-adherence sometimes difficult. The most common reason for compliancy difficulties was the restriction of favorite foods (76.2%, n = 16) (Fig. 3C). The subjects reporting unsuccessful dietary modification compliancy (25.7%, n = 34) most commonly cited perceived lack of improvement in their IBD symptoms as the primary reason for stopping the diet (48.4%, n = 15) (Fig. 3D).

### Disease Knowledge

We assessed the subject’s knowledge of their disease and its medical treatment as a means to determine if subjects modified their diet based upon their understanding of their disease. In our study, only 21% of subjects indicated that they understood their disease diagnosis enough to fully explain it in detail to others. Compared to EMR-R, 94.7% (n = 125) subjects correctly identified their IBD subtype with very good agreement between SR and EMR-R data (AC1 = 0.94, CI = 0.90, 0.98), but only 48.1% (n = 63) correctly identified disease location (AC1 = 0.33, CI = 0.22, 0.46) (Table 1). Additionally, 66.7% (n = 88) and 67.4% (n = 89) of subjects had good knowledge (>75%) of physician-prescribed medications (Supplementary Data Content 3A) and nutrition supplements (Supplementary Data Content 3B), respectively. Complete EMR-R medication usage is listed in Table 1. When comparing current diet users (n = 33) with nonusers (n = 99), we found no correlation between knowledge of prescribed medications and current use of diet modification (*P* = 0.67). Similarly, we found no correlation between prior utilization of diet modification (n = 34) and knowledge of prescribed medications (*P* = 0.32).

Interestingly, even though 81.8% (n = 108) of subjects had EMR-R dietary supplement recommendations, over half of subjects (64.4%, n = 85) indicated nonuse of supplements (n = 19, AC1 = 0.044, CI = −0.22, 0.14) (Table 1). Vitamin D was the highest prescribed dietary supplement, and merely 50.0% of subjects correctly identified it as being prescribed, resulting in poor agreement (AC1 = −0.044, CI = −0.22, 0.14). Most subjects (71.8%, n = 94) specified that they were at least partially in charge of ensuring they follow their prescribed treatment plan, with a near equal proportion (69.5%, n = 91) receiving assistance from their parent or guardian.

### Sources of Disease and Treatment Information

**Figure 4.**
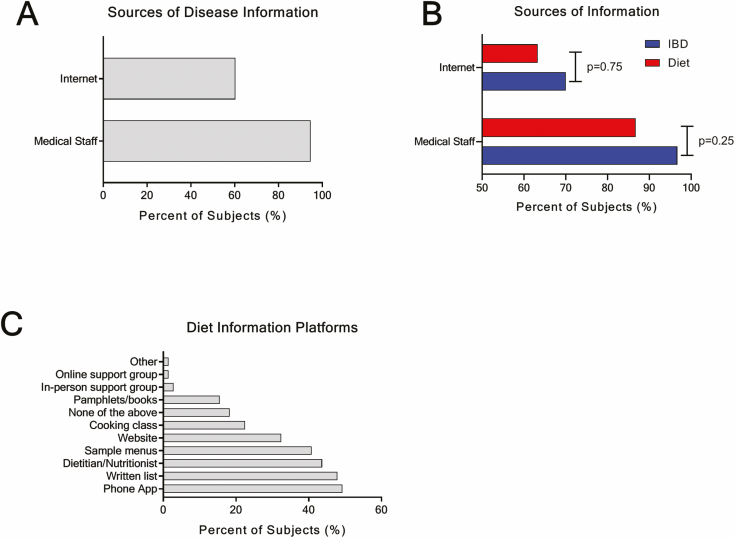
Sources of information. A, Breakdown of percent of subjects who talk to medical professionals (doctors, nurses, and dietitians) and use internet sources to gather information on IBD. B, Breakdown of current dietary modification users who gather information on IBD (blue) and IBD-related dietary modification protocols (red) from medical professionals and internet sources. McNemar’s test was used to assess differences in source of information utilization. C, Resources identified as being helpful when potentially trying a dietary modification protocol in the future.

Subjects had a high preference (94.7%, n = 124) for receiving information about their disease and medication options from medical professionals (doctors, nurses, and dietitians), and many subjects added to this with information they found from the internet (60.3%, n = 79) (Fig. 4A). Among subjects currently modifying their diet, there were no differences in what resources they used to collect information about disease and diet information. From medical professionals, 96.7% sought information about IBD, whereas 86.7% received information about potential diets to manage their disease symptoms (*P* = 0.25) (Fig. 4B). Similarly, 70.0% supplemented information about IBD with internet resources, and 63.3% turned to the internet for additional information on diets (*P* = 0.75) (Fig. 4B).

When subjects who had never tried modifying their diet (n = 71) were asked what resources they believed would make it easier for them to try dietary modification, most preferred to have a written list of approved food items (47.9%, n = 34), work directly with a dietitian/nutritionist (43.7%, n = 31), or be provided with sample menus (40.8%, n = 29) (Fig. 4C). Most of these subjects stated they preferred a web-based platform, such as a web site (32.4%, n = 23) or phone application with a searchable database (49.3%, n = 35), as opposed to written printed information in the form of a pamphlet or book (15.5%, n = 11) to help them garner information on dietary modifications (Fig. 4C).

## DISCUSSION

In this cross-sectional survey of adolescents and young adults with IBD, we determined that they not only identified a relationship between their diet and their disease symptoms, but that they self-modified their diet in an attempt to achieve symptom relief. While subjects who tried modifying their diet often found little success and were faced with a multitude of perceived compliance challenges, it was unclear whether this was due to the lack of efficacy for the (self)-selected dietary modifications, lack of guidance by a trained dietitian, or noncompliance. Diet is an important factor in both the onset and the progression of IBD,^5,8,27,28^ and global shifts in food consumption have been implicated in its increasing incidence.^29–31^ Surveys of adult IBD patients indicate that 48%–71% of patients believe that diet impacts their disease^21,32,33^; however, few studies have looked at whether adolescent patients share this belief, or are willing to try dietary modifications.^34^ What is clear from this study is that disease symptoms and patient-reported disease activity influence the decision of adolescents with IBD to self-modify their diet.

With the increase in knowledge of the pathophysiology of IBD, there has been significant interest in the relationship between diet and inflammation in IBD.^4^ Specifically, diet or individual dietary components have become a focal point of basic science and clinical research to ameliorate the intestinal inflammatory response in IBD. This is driven by both physician and patient preference to find safe, effective treatment options while minimizing side effects. Medical nutrition therapy provided by a registered dietitian–nutritionist has been proven a successful therapy in controlling other chronic diseases such as metabolic syndrome, cardiovascular disease, obesity, type II diabetes mellitus, and chronic kidney disease.^35–38^ In the treatment of Crohn’s disease exclusive enteral nutrition (EEN) is considered an optimal therapy to induce remission in children and adolescents due to the combination of low side effects and high efficacy (80%–85% remission rate).^7,9,39^ Implementation of EEN requires strict guidance and support from medical staff, and patients are largely restricted from consuming solid foods. Many dietary protocols have been developed in the hopes of recapitulating the efficacy of EEN but allowing for consumption of ordinary foods, albeit with specific restrictions. The specific carbohydrate (which was popular among subjects in our study), Crohn’s Disease Exclusion Diet, low-FODMAP (fermentable oligo-, di-, mono-saccharides, and polyols), an autoimmune protocol, and Anti-Inflammatory (IBD-AID) diets have been shown to illicit changes in the fecal microbiome and aid in the resolution of symptoms, but to date have not been shown to result in complete remission with endoscopic mucosal healing and normalization of inflammatory markers.^16,40–44^

As our data suggest, adherence to a stringent diet can be challenging due to a variety psychological, social, and dietary-restriction related reasons. Strict elimination diets can make it difficult to identify approved food items. Additionally, an individual’s favorite foods may not be on the approved list, while undesirable foods may be encouraged, decreasing enthusiasm to adhere to the diet. In society today, eating is closely tied to social interactions, and limitations in dietary choices can create both social and psychological strains.^14^ Stress, in turn, can have a negative impact on dietary choices and behaviors.^45,46^ Stress has been identified as a key IBD symptom trigger in both this study and others,^47^ and the complex relationship between stress and food can compound to further exacerbate IBD symptoms. Further research and efforts must be made to uncouple these factors as they may create a vicious cycle that may result in persistent aggravation of disease symptoms.

Technology is rapidly advancing, and the platforms in which adolescents prefer to receive information tend to be drastically different than prior generations. While our subjects still readily sought advice from medical staff, they also often turned to less reliable sources, such as internet blogs and web sites. This may be why our study identified that a large proportion of subjects were utilizing dietary modifications without documented physician and/or dietitian direction or support. This is a largely unreported phenomenon in pediatric IBD. Food avoidance is commonly documented, but attempts to self-adhere to a specified dietary modification are rarely reported.^34,48^ Many web sites contain reliable information with good-intentioned advice, but the information may be hard to understand and reliable sources may be overshadowed with more attractive and misleading information. The vast amount of readily available information may have contributed to both the desire to try “dietary freelancing” and the limited success with the dietary interventions experienced in our cohort.

The results of this survey are encouraging for expanding the study of diet-based therapy and promoting the willingness of physicians to implement in adolescent IBD populations. Even if the physician is not broaching the topic of diet, patients are interested and pursue dietary modification on their own. An open dialogue between patients and their gastroenterologist, along with frequent consultation with a dietitian, about dietary modification could help focus patients on a specified protocol that has the most potential to benefit them, and ensure they maintain compliance and adequate nutritional status.

A limitation of our study was the small, single-center study population, but many of our findings echo what has been determined in other studies—adolescent IBD patients perceive a link between diet and disease (59.8% in our study) and are receptive to diet therapy (45.4% in our study), similar to 7%–48% in other reports.^16,17,33,34,48^ While our study population was small, our response rate was robust at nearly 90%. This may be due to the use of an electronic tablet platform to administer the survey. Electronic and web-based platforms are highly popular with younger patients; physicians and researchers should readily take advantage of this when directing patients to reliable information sources or conducting future studies.

Our survey was designed to cast a broad net to collect information pertaining to a large variety of topics, including disease knowledge, perceptions on current treatments and the potential of dietary modification therapy, current dietary habits, and sources of information. While we were able to capture a large amount of information directly from adolescent IBD patients, we cannot draw any conclusions on the level of compliance and how that correlates to perceived success or failure of a dietary modification. We also surveyed patients, while the recommendations of medical staff were limited to EMR documentation.

## CONCLUSIONS

Dietary protocols to treat IBD and disease symptoms continue to be a growing area of research, but patient interest in this new potential avenue of therapy that requires significant effort and compliance is rarely reported. Our study suggests that a large proportion of adolescent IBD patients may already be attempting dietary modification, and therefore would be receptive to a modified dietary plan under the guidance of their gastroenterologist and dietitian. Much is still unknown about how dietary modification will fit in with current treatment regimens, but patient interest informs us that it is necessary to continue development and research of this promising therapeutic option.

## Supplementary Material

otaa084_suppl_Supplementary_Materials_1

## Data Availability

The authors confirm that the data supporting the findings of this study are available within the article and/or its Supplementary Material. Survey tool materials are available upon request.

## Notes

Funding: This study was supported by a grant from the Cleveland Clinic Research Programs Committees Pilot Program (#259 to M.T.Z.) and the philanthropic support of CURE4IBD (to C.M.).

## References

[1] Cosnes J, Gower-Rousseau C, Seksik P, et al. Epidemiology and natural history of inflammatory bowel diseases. Gastroenterology. 2011;140:1785–1794. 21530745 10.1053/j.gastro.2011.01.055

[2] Benchimol EI, Fortinsky KJ, Gozdyra P, et al. Epidemiology of pediatric inflammatory bowel disease: a systematic review of international trends. Inflamm Bowel Dis. 2011;17:423–439. 20564651 10.1002/ibd.21349

[3] Rogers BH, Clark LM, Kirsner JB. The epidemiologic and demographic characteristics of inflammatory bowel disease: an analysis of a computerized file of 1400 patients. J Chronic Dis. 1971;24:743–773. 5146188 10.1016/0021-9681(71)90087-7

[4] Lewis JD, Abreu MT. Diet as a trigger or therapy for inflammatory bowel diseases. Gastroenterology. 2017;152:398–414.e6. 27793606 10.1053/j.gastro.2016.10.019

[5] Khalili H, Chan SSM, Lochhead P, et al. The role of diet in the aetiopathogenesis of inflammatory bowel disease. Nat Rev Gastroenterol Hepatol. 2018;15:525–535. 29789682 10.1038/s41575-018-0022-9 PMC6397648

[6] Aleksandrova K, Romero-Mosquera B, Hernandez V. Diet, gut microbiome and epigenetics: emerging links with inflammatory bowel diseases and prospects for management and prevention. Nutrients. 2017;9:962. 28867793 10.3390/nu9090962 PMC5622722

[7] Levine A, Sigall Boneh R, Wine E. Evolving role of diet in the pathogenesis and treatment of inflammatory bowel diseases. Gut. 2018;67:1726–1738. 29777041 10.1136/gutjnl-2017-315866

[8] Penagini F, Dilillo D, Borsani B, et al. Nutrition in pediatric inflammatory bowel disease: from etiology to treatment. A systematic review. Nutrients. 2016;8:334. 27258308 10.3390/nu8060334 PMC4924175

[9] Hansen T, Duerksen DR. Enteral nutrition in the management of pediatric and adult Crohn’s disease. Nutrients. 2018;10:537. 29701656 10.3390/nu10050537 PMC5986417

[10] Kaenkumchorn T, Kesavan A. Dietary management of pediatric inflammatory bowel disease. J Med Food. 2019;22:1092–1099. 31329006 10.1089/jmf.2019.0063

[11] Lane ER, Lee D, Suskind DL. Dietary therapies in pediatric inflammatory bowel disease: an evolving inflammatory bowel disease paradigm. Gastroenterol Clin North Am. 2017;46:731–744. 29173518 10.1016/j.gtc.2017.08.012

[12] Lewis JD, Chen EZ, Baldassano RN, et al. Inflammation, antibiotics, and diet as environmental stressors of the gut microbiome in pediatric Crohn’s disease. Cell Host Microbe. 2015;18:489–500. 26468751 10.1016/j.chom.2015.09.008 PMC4633303

[13] Taddeo D, Egedy M, Frappier JY. Adherence to treatment in adolescents. Paediatr Child Health. 2008;13:19–24. 19119348 10.1093/pch/13.1.19 PMC2528818

[14] Czuber-Dochan W, Morgan M, Hughes LD, et al. Perceptions and psychosocial impact of food, nutrition, eating and drinking in people with inflammatory bowel disease: a qualitative investigation of food-related quality of life. J Hum Nutr Diet. 2020;33:115–127. 31131484 10.1111/jhn.12668

[15] Brenton JN, Goldman MD. A study of dietary modification: perceptions and attitudes of patients with multiple sclerosis. Mult Scler Relat Disord. 2016;8:54–57. 27456874 10.1016/j.msard.2016.04.009

[16] Suskind DL, Wahbeh G, Cohen SA, et al. Patients perceive clinical benefit with the specific carbohydrate diet for inflammatory bowel disease. Dig Dis Sci. 2016;61:3255–3260. 27638834 10.1007/s10620-016-4307-y

[17] Obih C, Wahbeh G, Lee D, et al. Specific carbohydrate diet for pediatric inflammatory bowel disease in clinical practice within an academic IBD center. Nutrition. 2016;32:418–425. 26655069 10.1016/j.nut.2015.08.025

[18] Ware JE Jr, Sherbourne CD. The MOS 36-item short-form health survey (SF-36). I. Conceptual framework and item selection. Med Care. 1992;30:473–483. 1593914

[19] Clara I, Lix LM, Walker JR, et al. The Manitoba IBD Index: evidence for a new and simple indicator of IBD activity. Am J Gastroenterol. 2009;104:1754–1763. 19455122 10.1038/ajg.2009.197

[20] Gwet KL . Computing inter-rater reliability and its variance in the presence of high agreement. Br J Math Stat Psychol. 2008;61:29–48. 18482474 10.1348/000711006X126600

[21] Limdi JK, Aggarwal D, McLaughlin JT. Dietary practices and beliefs in patients with inflammatory bowel disease. Inflamm Bowel Dis. 2016;22:164–170. 26383912 10.1097/MIB.0000000000000585

[22] Kinsey L, Burden S. A survey of people with inflammatory bowel disease to investigate their views of food and nutritional issues. Eur J Clin Nutr. 2016;70:852–854. 27117934 10.1038/ejcn.2016.57

[23] Larussa T, Suraci E, Marasco R, et al. Self-prescribed dietary restrictions are common in inflammatory bowel disease patients and are associated with low bone mineralization. Medicina (Kaunas). 2019;55:507. 31434334 10.3390/medicina55080507 PMC6722983

[24] Vagianos K, Clara I, Carr R, et al. What are adults with inflammatory bowel disease (IBD) eating? A closer look at the dietary habits of a population-based Canadian IBD cohort. JPEN J Parenter Enteral Nutr. 2016;40:405–411. 25189173 10.1177/0148607114549254

[25] Vidarsdottir JB, Johannsdottir SE, Thorsdottir I, et al. A cross-sectional study on nutrient intake and -status in inflammatory bowel disease patients. Nutr J. 2016;15:61. 27268004 10.1186/s12937-016-0178-5 PMC4897945

[26] Zallot C, Quilliot D, Chevaux JB, et al. Dietary beliefs and behavior among inflammatory bowel disease patients. Inflamm Bowel Dis. 2013;19:66–72. 22467242 10.1002/ibd.22965

[27] Uranga JA, López-Miranda V, Lombó F, et al. Food, nutrients and nutraceuticals affecting the course of inflammatory bowel disease. Pharmacol Rep. 2016;68:816–826. 27267792 10.1016/j.pharep.2016.05.002

[28] Green N, Miller T, Suskind D, et al. A review of dietary therapy for IBD and a vision for the future. Nutrients. 2019;11:947. 31035465 10.3390/nu11050947 PMC6566428

[29] Rizzello F, Spisni E, Giovanardi E, et al. Implications of the westernized diet in the onset and progression of IBD. Nutrients. 2019;11:1033. 31072001 10.3390/nu11051033 PMC6566788

[30] Chiba M, Nakane K, Komatsu M. Westernized diet is the most ubiquitous environmental factor in inflammatory bowel disease. Perm J. 2019;23:18–107. 10.7812/TPP/18-107 PMC6326567 30624192

[31] Yang Y, Owyang C, Wu GD. East Meets West: the increasing incidence of inflammatory bowel disease in Asia as a paradigm for environmental effects on the pathogenesis of immune-mediated disease. Gastroenterology. 2016;151:e1–e5. 10.1053/j.gastro.2016.10.034 PMC5439349 27810482

[32] Holt DQ, Strauss BJ, Moore GT. Patients with inflammatory bowel disease and their treating clinicians have different views regarding diet. J Hum Nutr Diet. 2017;30:66–72. 27412965 10.1111/jhn.12400

[33] de Vries JHM, Dijkhuizen M, Tap P, et al. Patient’s dietary beliefs and behaviours in inflammatory bowel disease. Dig Dis. 2019;37:131–139. 30391940 10.1159/000494022 PMC6381876

[34] Pituch-Zdanowska A, Kowalska-Duplaga K, Jarocka-Cyrta E, et al. Dietary beliefs and behaviors among parents of children with inflammatory bwel disease. J Med Food. 2019;22:817–822. 31063436 10.1089/jmf.2018.0206

[35] Perry RA, Daniels L, Baur LA, et al. Impact of a 6-month family-based weight management programme on child food and activity behaviours: short-term and long-term outcomes of the PEACH™ intervention. Pediatr Obes. 2018;13:744–751. 30280513 10.1111/ijpo.12460

[36] Cole HS, Camerini-Davalos RA. Diet therapy of diabetes mellitus. Med Clin North Am. 1970;54:1577–1587. 5487682

[37] Connor H, Annan F, Bunn E, et al.; Nutrition Subcommittee of the Diabetes Care Advisory Committee of Diabetes UK. The implementation of nutritional advice for people with diabetes. Diabet Med. 2003;20:786–807. 14510859 10.1046/j.1464-5491.2003.01104.x

[38] Anderson KL . A review of the prevention and medical management of childhood obesity. Child Adolesc Psychiatr Clin N Am. 2018;27:63–76. 29157503 10.1016/j.chc.2017.08.003

[39] Bishop J, Lemberg DA, Day A. Managing inflammatory bowel disease in adolescent patients. Adolesc Health Med Ther. 2014;5:1–13. 24729736 10.2147/AHMT.S37956 PMC3956483

[40] Suskind DL, Cohen SA, Brittnacher MJ, et al. Clinical and fecal microbial changes with diet therapy in active inflammatory bowel disease. J Clin Gastroenterol. 2018;52:155–163. 28030510 10.1097/MCG.0000000000000772 PMC5484760

[41] Cox SR, Lindsay JO, Fromentin S, et al. Effects of low FODMAP diet on symptoms, fecal microbiome, and markers of inflammation in patients with quiescent inflammatory bowel disease in a randomized trial. Gastroenterology. 2020;158:176–188.e7. 31586453 10.1053/j.gastro.2019.09.024

[42] Olendzki BC, Silverstein TD, Persuitte GM, et al. An anti-inflammatory diet as treatment for inflammatory bowel disease: a case series report. Nutr J. 2014;13:5. 24428901 10.1186/1475-2891-13-5 PMC3896778

[43] Chandrasekaran A, Groven S, Lewis JD, et al. An autoimmune protocol diet improves patient-reported quality of life in inflammatory bowel disease. Crohns Colitis 360. 2019;1:otz019. 31832627 10.1093/crocol/otz019 PMC6892563

[44] Levine A, Wine E, Assa A, et al. Crohn’s disease exclusion diet plus partial enteral nutrition induces sustained remission in a randomized controlled trial. Gastroenterology. 2019;157:440–450.e8. 31170412 10.1053/j.gastro.2019.04.021

[45] Nastaskin RS, Fiocco AJ. A survey of diet self-efficacy and food intake in students with high and low perceived stress. Nutr J. 2015;14:42. 25902797 10.1186/s12937-015-0026-z PMC4416420

[46] Errisuriz VL, Pasch KE, Perry CL. Perceived stress and dietary choices: the moderating role of stress management. Eat Behav. 2016;22:211–216. 27310611 10.1016/j.eatbeh.2016.06.008

[47] Mawdsley JE, Rampton DS. The role of psychological stress in inflammatory bowel disease. Neuroimmunomodulation. 2006;13:327–336. 17709955 10.1159/000104861

[48] Chuong KH, Haw J, Stintzi A, et al. Dietary strategies and food practices of pediatric patients, and their parents, living with inflammatory bowel disease: a qualitative interview study. Int J Qual Stud Health Well-Being. 2019;14:1648945. 31382870 10.1080/17482631.2019.1648945 PMC6713182

