# Supplementary material for: Impact of Diet on Inflammatory Bowel Disease Symptoms: An Adolescent Viewpoint
Source: Crohns Colitis 360. 2020 Nov 12;2(4):otaa084. doi: 10.1093/crocol/otaa084 (PMC9802055; doi:10.1093/crocol/otaa084)
Supplement: otaa084_suppl_Supplementary_Materials_1 [file otaa084_suppl_supplementary_materials_1.pdf]

Supplementary Data Content 1

	Never	Less than once per week	1-3 days per week	4-6 days per week	Every day	Don't Know
3 meals per day	1 (0.76)	12 (9.2)	18 (13.7)	25 (19.1)	73 (55.7)	2 (1.5)
Sports drinks	40 (30.8)	40 (30.8)	31 (23.8)	8 (6.2)	9 (6.9)	2 (1.5)
Fresh fruit	8 (6.1)	18 (13.7)	36 (27.5)	32 (24.4)	36 (27.5)	1 (0.76)
Milk products	9 (7.0)	13 (10.1)	27 (20.9)	24 (18.6)	55 (42.6)	1 (0.78)
Pop/soda	33 (25.4)	41 (31.5)	32 (24.6)	9 (6.9)	15 (11.5)	0 (0)
Bread/grains	6 (4.6)	4 (3.1)	21 (16.0)	36 (27.5)	64 (48.9)	0 (0)
Condiments/sauces	5 (3.8)	16 (12.3)	39 (30.0)	37 (28.5)	30 (23.1)	3 (2.3)
Caffeine	37 (28.2)	28 (21.4)	22 (16.8)	17 (13.0)	24 (18.3)	3 (2.3)
Red meat	17 (13.0)	22 (16.8)	53 (40.5)	35 (26.7)	2 (1.5)	2 (1.5)
Legumes/beans	36 (27.5)	42 (32.1)	36 (27.5)	8 (6.1)	6 (4.6)	3 (2.3)
Sweets/Dessert	7 (5.3)	32 (24.4)	45 (34.4)	29 (22.1)	16 (12.2)	2 (1.5)
Nuts/seeds	42 (32.1)	41 (31.3)	30 (22.9)	14 (10.7)	1 (0.76)	3 (2.3)
Fast food	14 (10.7)	57 (43.5)	43 (32.8)	11 (8.4)	5 (3.8)	1 (0.76)
Eggs	14 (10.8)	29 (22.3)	49 (37.7)	28 (21.5)	9 (6.9)	1 (0.77)
Fruit juice	21 (16.2)	36 (27.7)	39 (30.0)	21 (16.2)	12 (9.2)	1 (0.77)
Processed foods	5 (3.8)	28 (21.4)	47 (35.9)	31 (23.7)	17 (13.0)	3 (2.3)
Snack/granola bars	11 (8.5)	18 (13.8)	46 (35.4)	33 (25.4)	21 (16.2)	1 (0.77)
Fresh vegetables	12 (9.2)	13 (9.9)	40 (30.5)	40 (30.5)	25 (19.1)	1 (0.76)
Lean meat	9 (6.9)	13 (9.9)	38 (29.0)	46 (35.1)	19 (14.5)	6 (4.6)
Restaurant food	8 (6.1)	54 (41.2)	47 (35.9)	13 (9.9)	7 (5.3)	2 (1.5)

Supplementary Data Content 1. Food frequency questionnaire. Answers represented as number of responses (percent of answers).

Supplementary Data Content 2

Category	Count	Items
Oily/fatty/greasy foods	22	Pizza, fried foods, fast food
Dairy & whey	21	
Spicy foods	18	
Fresh fruits & vegetables	15	Cabbage, lettuce, salads, leafy vegetables, onions, strawberries, carrots, celery
Acidic foods	10	Tomato products, red sauces
Seeds & nuts	10	Raw nuts, sesame seeds, poppy seeds, peanuts
Starch	8	Corn, beans
Sweets	7	Sugary foods, apple juice
Popcorn	7	
Preservatives/processed foods	6	Preservatives, processed foods, “junk food,” chips
Fiber	6	
Gluten	5	
Red meat	5	Pork, beef
Caffeine	4	Coffee
Chocolate	3	
Fructose	2	
Whole grains	2	Wheat
Eggs	1	
Alcohol	1	Beer
Chicken	1	

Supplementary Data Content 2. Complete list of food categories identified by subjects as potential IBD symptoms triggers. Subjects who selected diet as a potential disease symptom trigger were given a fillable text box and asked to identify any and all foods/products/food characteristics they believe trigger disease symptoms. Responses were grouped into categories based on likeness of the food product or description. Items combined into broader categories are listed individually in “items” column.

Supplementary Data Content 3

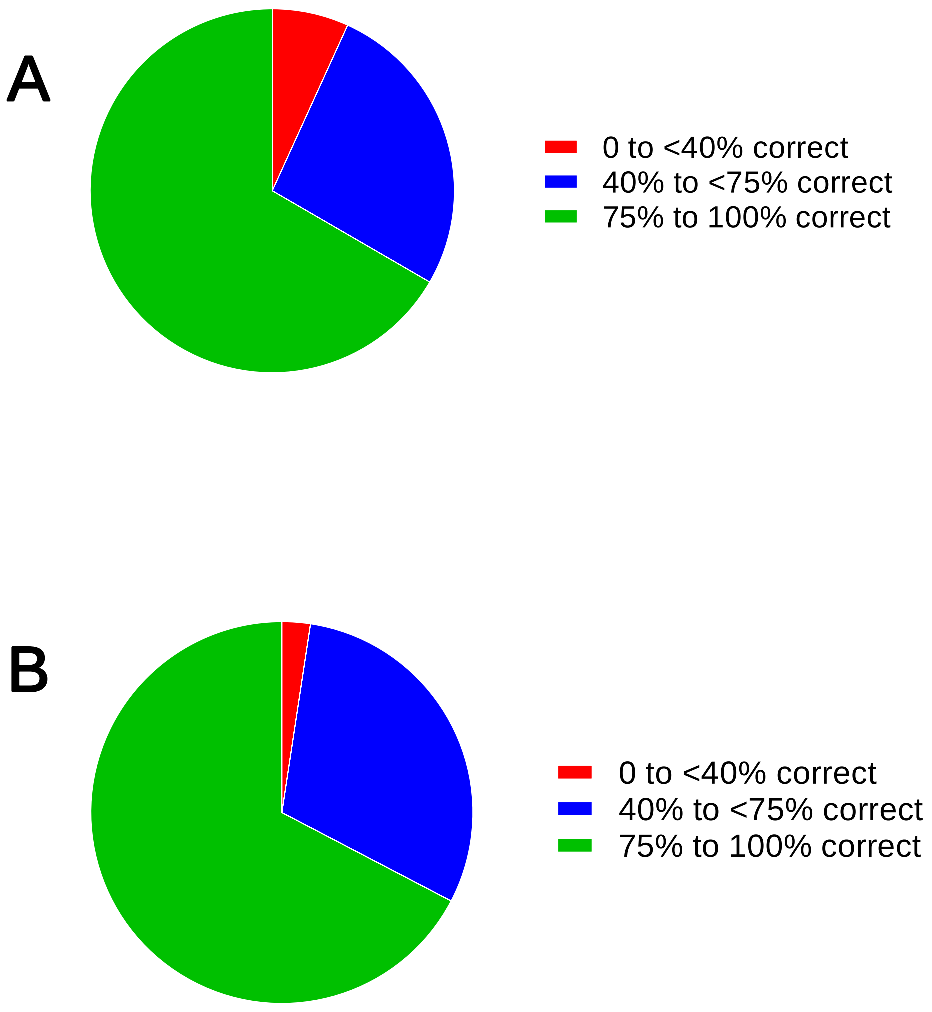

Supplementary Data Content 3. Subject knowledge of (A) prescribed medications and (B) recommended dietary supplements. The ability to identify correct answers and leave incorrect answers blank was assessed using a multi-select option list. Selecting greater than 75% of correct answers (green) was considered good knowledge, between 40% and 75% (blue) fair knowledge, and less than 40% (red) poor knowledge.
